# There are unique kinematics during locomotor transitions between level ground and stair ambulation that persist with increasing stair grade

**DOI:** 10.1038/s41598-023-34857-7

**Published:** 2023-05-26

**Authors:** Ross M. Neuman, Nicholas P. Fey

**Affiliations:** Walker Department of Mechanical Engineering, The University of Texas at Austin, 204 E Dean Keeton St, Austin, TX 78712 USA

**Keywords:** Biomedical engineering, Translational research

## Abstract

Human ambulation is typically characterized during steady-state isolated tasks (e.g., walking, running, stair ambulation). However, general human locomotion comprises continuous adaptation to the varied terrains encountered during activities of daily life. To fill an important gap in knowledge that may lead to improved therapeutic and device interventions for mobility-impaired individuals, it is vital to identify how the mechanics of individuals change as they transition between different ambulatory tasks, and as they encounter terrains of differing severity. In this work, we study lower-limb joint kinematics during the transitions between level walking and stair ascent and descent over a range of stair inclination angles. Using statistical parametric mapping, we identify where and when the kinematics of transitions are unique from the adjacent steady-state tasks. Results show unique transition kinematics primarily in the swing phase, which are sensitive to stair inclination. We also train Gaussian process regression models for each joint to predict joint angles given the gait phase, stair inclination, and ambulation context (transition type, ascent/descent), demonstrating a mathematical modeling approach that successfully incorporates terrain transitions and severity. The results of this work further our understanding of transitory human biomechanics and motivate the incorporation of transition-specific control models into mobility-assistive technology.

## Introduction

Studies involving human mobility often isolate a particular task of interest, such as level walking or inclined walking, and attempt to capture the steady-state biomechanics driving that task. In contrast, general human ambulation involves continuous adjustment to the varying terrain one may encounter while moving from one place to the next. In order to better understand human movement, it is necessary to investigate the mechanics that enable successful transitions between these terrains and account for the severity of a given terrain.

The tasks of ascending and descending the steps of a staircase show the importance of adapting to varied terrain. Falls occurring on stairs present a significant public health risk to people of all ages^1–3^, are responsible for over 1,000,000 visits to United States emergency departments annually^4^, and represent a major cause of traumatic brain injury in adults^2^. Stairs can be particularly hazardous for the elderly, who must contend with age-related changes in mobility^5–8^ and are more likely to experience potentially-fatal bone fractures following a fall^1,3^. There is also a wide range of allowable staircase dimensions within building regulations^9^ which can be a confounder in studies about stair falls^10^, furthering the difficulty of assessing fall hazards in a standardized manner^11^. With many falls occur during the transitions between level ground and stair walking^12^, the mechanics employed during these transitionary phases are relevant to understanding these incidents.

Further motivation can be found in the area of powered mobility-assistive devices, where considerable research has focused on of how best to handle the transitions between level ground and stairs. Strategies range from the simple methods of user-operated switching^13^, to volitional switching with EMG^14–16^, anticipatory strategies based on input from biomechanical sensors^17,18^, and more recently computer vision^19,20^. To successfully emulate the fluency of able-bodied ambulation, an assistive device would ideally facilitate not only the timely transition between level walking and stair walking, but also the mechanics of the transition between the two terrains^21^. This was demonstrated in a recent study^22^ that modeled transition kinematics by interpolating between steady-state modes and incorporating an additional term to account for kinematics only seen in transitions, operating on the assumption that transition kinematics require special treatment from a modeling perspective. Thus, identifying whether transition kinematics are unique and how they deviate from those of steady-state walking will supplement and motivate the continued refinement of assistive technologies.

Prior studies about stair biomechanics have noted that individuals share some basic mechanical patterns^23^ when traversing stairs, and that stair inclination angle affects kinematics and kinetics in the lower limbs^24,25^. Others have observed that the transitions between level walking and stair walking are accompanied by anticipatory mechanics and muscle activations^26,27^. While these studies demonstrate that there are biomechanical responses elicited by transitions and terrain severity in stair walking, there are presently no studies that statistically characterize how and when the lower limb mechanics change under these circumstances.

In this study, we highlight the differences in sagittal-plane lower-limb joint trajectories during the locomotor transitions between level walking and stair walking, as well as the effects of inclination angle on stair walking mechanics. This includes the transitions into and out of stair walking for both ascending and descending tasks, with these tasks repeated over four different inclination angles. Kinematic data collected from ten able-bodied subjects^28^ was separated into level walking (LW), transition (TR), and stair walking (SW) strides. Using statistical parametric mapping (SPM), we compare full-stride joint trajectories against each other rather than specific points of interest, for a more holistic picture of how joint kinematics vary due to these contextual and environmental factors. We hypothesize that transition strides will present unique joint kinematics when compared to those of level walking and stair walking. Furthermore, we hypothesize that the angle of staircase inclination will affect kinematics in transition strides, despite the modest difference in stair height between inclinations.

Finally, we perform a Gaussian process regression (GPR) on the same dataset to predict joint angles based on continuous gait phase and stair grade as well as discrete ambulation context (e.g., ascending/descending, transition type). GPR is a “black-box” statistical method that has been used in gait-related contexts^29–31^ for its ability to handle the highly nonlinear joint trajectories and to incorporate the variability of the underlying dataset^32^. Because transitions represent an interruption to the regularity enforced by evenly-spaced stairs or steady-speed treadmill walking, we anticipate seeing higher variability and therefore higher model error for transition kinematics. Using cross-validation to assess the model error for each joint over each task, we hypothesize that there will be higher error in predictions of transition kinematics compared steady-state tasks.

## Results

**Figure 1:**
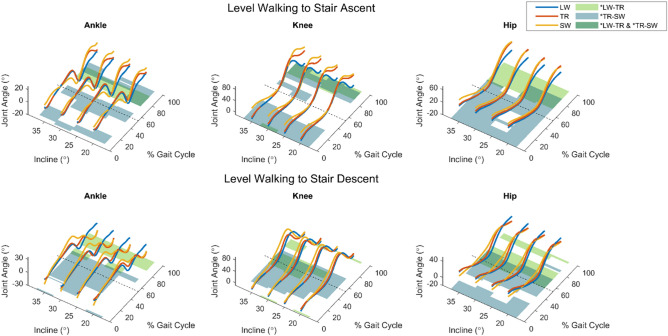
Results from SPM analysis of walk-to-stair transitions. Solid lines indicate the mean joint angle trajectories of the $n=10$ subjects for level walking (LW), walk-to-stair transitions (TR), and stair walking (SW), plotted over a full gait cycle. Foot-off is indicated by the dotted line. Shaded regions indicate statistically significant ($p<0.05$) differences between stride types as determined by paired SPM t-tests. Green regions indicate differences between level walking and transition strides, while blue regions represent differences between transitions and stair walking. Areas of overlap between the blue and green regions appear darker green, and represent the areas where transition kinematics are considered unique from either steady-state task.

**Figure 2:**
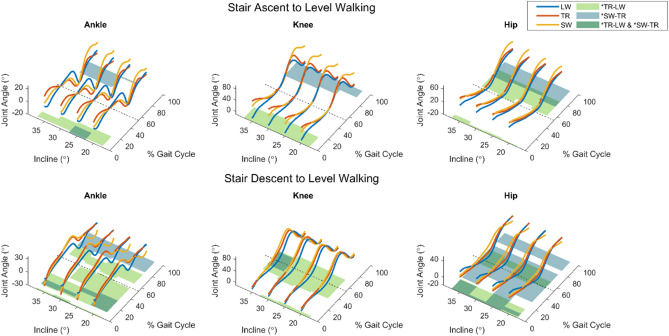
Results from SPM analysis of stair-to-walk transitions. Solid lines indicate the mean joint angle trajectories of the subjects for level walking (LW), walk-to-stair transitions (TR), and stair walking (SW), plotted over a full gait cycle. Foot-off is indicated by the dotted line. For the stair descent to level walking results, $n=10$ subjects, and for the stair ascent to level walking results, $n=6$ subjects. Shaded regions indicate statistically significant ($p<0.05$) differences between stride types as determined by paired SPM t-tests. Green regions indicate differences between level walking and transition strides, while blue regions represent differences between transitions and stair walking. Areas of overlap between the blue and green regions appear darker green, and represent the areas where transition kinematics are considered unique from either steady-state task.

The following figures show the results obtained from SPM analyses of entire strides. For more information please see the methods section. Shaded areas behind the joint trajectories indicate regions of statistically significant difference between trajectories. Regions of difference between level walking and transition trajectories (LW–TR) and between transition and stair walking trajectories (TR–SW) are shown in the shaded regions of Figs. 1 and 2. In support of our hypothesis, regions of the TR trajectories were significantly different from those of LW and SW for every joint in every condition. Overlapping shaded regions indicate portions of TR trajectories that are statistically unique from LW *and* SW.

### Level walking to stair ascent

At the ankle, LW–TR significance appeared during mid-swing while TR–SW significance appeared in three distinct regions: early stance, late stance, and late swing. In the knee and hip, LW–TR significance appeared in mid-to-late stance. Hip TR–SW significance was generally present for all of stance and into early swing, while for the knee TR–SW was significant in early stance and various portions of swing. Significance did not appear related to the inclination angle.

### Level walking to stair descent

At the ankle, LW–TR significance appeared during late swing while TR–SW was significant during mid-late stance and early swing. At the knee, LW–TR significant differences were observed around the transition from stance to swing, though this was not observed at the 20° incline. Knee TR–SW differences were seen in a consistent, contiguous region from mid-late stance through early swing. At the hip, LW–TR significance was seen in early swing and late swing, while TR–SW significance occurred in early stance and late stance to early swing. TR–SW regions of significance in the ankle and knee tended increase with increasing inclination.

### Stair ascent to level walking

TR–LW significance was observed in early stance for every joint. For the hip joint, TR–LW significance was also present in early-to-mid swing. SW-TR significance occurred in late swing for all joints, and additionally during early stance at the ankle for one inclination. Higher inclination angles saw more SW-TR significance in the ankle and knee, and less TR–LW significance at the ankle.

### Stair descent to level walking

At the ankle, TR–LW differences were present in large regions spanning from mid stance to mid swing and SW-TR differences were seen in mid stance and late swing. TR–LW differences in the knee were observed from late stance through early swing. Very few regions of significant difference in SW-TR at the knee were observed, showing a sustained duration only in the 35° condition. For the hip, LW–TR differences were present in early stance and from late stance to early swing, while TR–LW differences spanned from mid stance through terminal swing.

### Effects of stair incline

**Figure 3:**
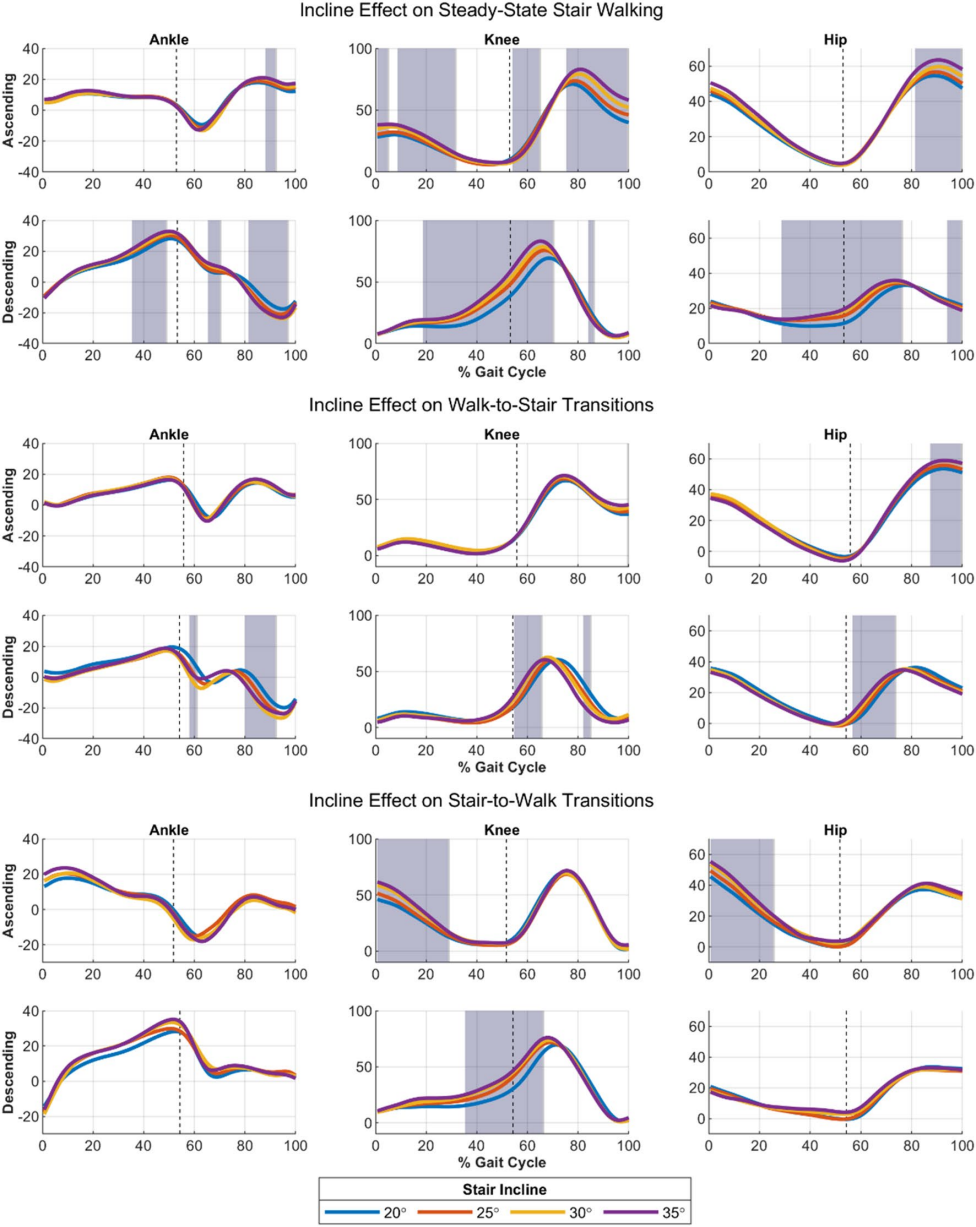
Effects of incline angle on stair walking tasks. Solid lines indicate mean joint angle trajectories averaged across all subjects for a complete gait cycle. Average foot-off for each task is indicated by the vertical dotted line. Shaded regions indicate significant $p<0.05$ main effects due to stair incline as determined by an SPM 1-way ANOVA.

Statistical Parametric Mapping (SPM) ANOVAs were performed exploring the effects of stair incline on SW and TR strides, and results are shown in Fig. 3. Here, shaded regions indicate main effects due to stair incline. While inclination angle affected at least one joint in all stride types, the majority of significant effects were observed in SW trajectories. Knee angles in particular showed effects due to incline over much of the gait cycle.

### Gaussian process regression surface fitting

A Gaussian process regression (GPR) model was trained on the collected data to allow continuous prediction of a joint angle based on the gait phase, inclination angle, direction of travel (ascending/descending), and ambulation task (steady-state stair, walk-to-stair, stair-to-walk). The root mean square error (RMSE) of model predictions based on leave-one-stride-out cross-validation is reported, and one-way ANOVAs determined that the ambulation task significantly (p < 0.05) affected the model error only for the ankle joint during stair ascent, where the stair-to-walk error was greater than both walk-to-stair and steady-state errors. Errors were largest in the knee joint and smallest in the ankle joint for all tasks.

**Figure 4:**
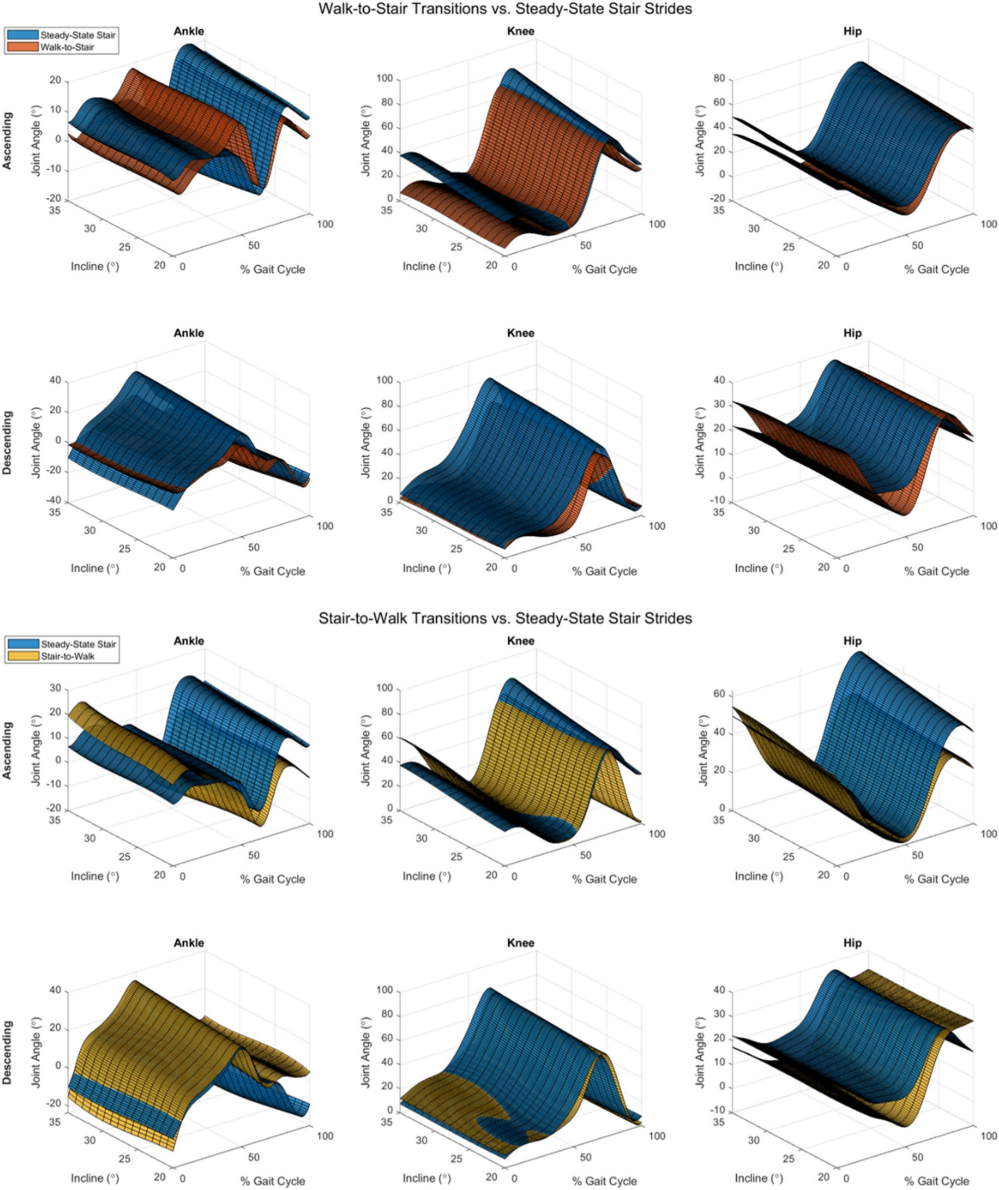
(Top) Comparison of joint angles predicted by Gaussian process regression models of steady-state stair and walk-to stair tasks across all inclines. (Bottom) Surfaces comparing predicted joint angles in steady-state stair and stair-to-walk tasks. Surfaces represent the expected value of the Gaussian process given the incline, gait phase, and task.

**Figure 5:**
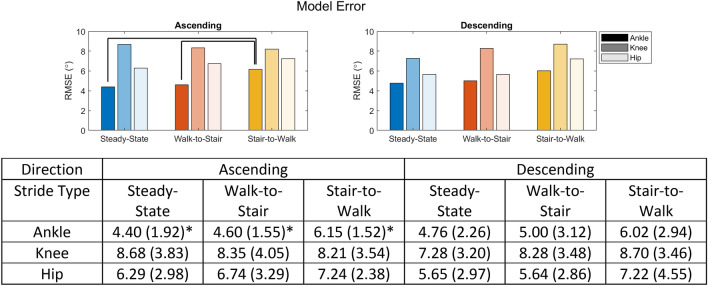
(Top) Model error (RMSE) in degrees for Gaussian process regressions based on leave-one-stride-out cross validation. Black lines indicate significant ($p<0.05$) pairwise difference between means as determined by a Tukey–Kramer test. Ankle, knee, and hip joints are grouped by color and differentiated by shading according to the legend. (Bottom) Model errors given as mean (standard deviation) in degrees. (*) indicates that there were significant ($p<0.05$) effects on model error due to stride type according to a 1-way ANOVA.

Using the GPR models, we plotted the predicted joint kinematics as a continuous surface over gait phase and stair inclination (Fig. 4). These plots help visualize the trends seen in Fig. 3. With increasing inclination angle, the peak knee flexion, hip flexion, dorsiflexion, and plantarflexion angles increased for steady-state stair walking. In walk-to-stair transitions, however, inclination angle had little effect on knee or ankle angles, while hip angles were still influenced. In stair-to-walk transitions, increasing inclination again caused general increases in peak angles for all joints, however the knee and hip saw changes during early stance rather than mid-late stance during ascending tasks. Leave-one-stride-out cross validation results (Fig. 5) showed that model errors were largely unaffected by the task, with only the ascending ankle tasks showing significant main effects, and pairwise comparisons indicating this was due to the error being significantly higher in the stair-to-walk task in this subset. In general, these results run counter to our hypothesis that the error would be higher during transition tasks.

## Discussion

In this study, we demonstrate the uniqueness of lower-limb joint kinematics during the transition strides between level walking and stair walking in able-bodied adults. Furthermore, we identify the specific regions of the gait cycle where TR strides differed from those of LW and SW. Visually, the kinematics show that TR trajectories tend to form a “hybrid stride” that closely tracks the previous ambulation mode early in the gait cycle, then tracks the following ambulation mode by the end of the cycle. While this pattern makes intuitive sense—TR strides facilitate a change from one steady state to the next—the findings presented here represent the first to quantify the way these changes occur. By statistically identifying the regions of the gait cycle over which kinematics differ for each joint, we are able to explore the details of when, where, and to what extent these broader transitory patterns hold true. The existence of unique transition kinematics demonstrates the continuously-adapting nature of general human locomotion and provides valuable insight and reference material for the developers of mobility-assistive technology. We also demonstrate that joint kinematics can be predicted with reasonable accuracy by a Gaussian process regression model given the gait phase, stair inclination angle, direction of travel, and stride type.

### Level walking to stair transitions

The transition from level walking to stair descent is perhaps the most crucial of the four transitions presented, given the injury potential from experiencing a fall at the top of a staircase. In this task, ankle TR trajectories (Fig. 1) provide a clear example of where abruptly changing from LW to SW is not ideal. While TR and LW are nearly identical from 0 to 60% GC, there is a sharp divergence around 80% GC, after which TR and SW are closely aligned for the remainder of the stride. In this region of late stance, TR and SW strides show a large plantarflexion peak as subjects prepare to make forefoot contact with the following stair^25^, where the plantarflexors are active in controlling the progression velocity^33^. While comprising only a small portion of the TR stride, this quick switch in ankle kinematic strategy may be crucial to safely transition from level walking to stair descent. Hip trajectories also show a departure from LW in late stance for this transition, extending earlier to make foot contact with a lower surface.

The transition into stair ascent also presents a fall risk, particularly in the case that the foot fails to clear the initial step. Furthermore, the forces experienced by the joints upon contact with this initial step can be far higher than in level walking^34^. Our data again suggest that neither LW nor SW trajectories would be sufficient substitutes for the TR stride: not only is the vertical displacement between consecutive foot contacts half of that in SW, but the horizontal distance is subject to vary substantially depending on the subject’s approach to the stairs^27^. The knee trajectories reflect this intermediate step height, as swing-phase TR angles fall between LW and SW by a large enough margin to register as unique. Swing-phase uniqueness is present at the ankle as well: rather than simply switching to resemble SW following the plantarflexion peak prior to toe-off, ankle TR strides plantarflex at mid-swing and terminate at an angle much closer to zero (horizontal foot placement) than is seen in SW. This behavior might serve to address the newly-encountered higher terrain by first dorsiflexing to insure foot clearance and aid in propulsion^35,36^, and then plantarflexing early to meet the level of the initial stair with the forefoot^37^. The hip trajectories for this transition into stair ascent are a clear example of a hybrid stride, where the strategy seems to switch from level walk to stair ascent upon switching from stance to swing. This is consistent with past findings about anticipatory adjustments for accommodating level changes^38^.

### Stair walking to level transitions

Moving from stair descent to level walking has been reportedly difficult for elderly populations^6^. In our statistical analysis, we again observed the trend where ankle TR strides form a hybrid of SW and LW, with a period of unique kinematics around the time of swing initiation. Meanwhile, TR trajectories at the knee show little to no difference from SW, with compensation for the transitions occurring instead at the hip. The hip joint TR achieves greater peak extension than SW, potentially indicating a longer step length as the leading leg lands on level ground, and then assumes LW-like swing mechanics with flexion lasting through foot contact.

Transitions from stair ascent to level walking, notably, were the only task of the four in which ankle TR trajectories were significantly different from the preceding mode during early stance, presenting with a large amount of dorsiflexion just after foot contact. This is also the only example of a peak ankle TR angle that is consistently greater than either SW or LW. This could again be preparing the leading leg to take a larger stride than preceding SW strides given the lack of step placement constraints imposed by stairs. The similar trend seen at the knee, with greater early stance flexion in TR than in SW or LW, corroborates this idea—if the TR stride is longer than SW, but still needs to cover a vertical distance of one stair, then the ankle and knee must undergo greater flexion to allow the leading leg to plant at the desired location.

### Implications for device control

The kinematics observed in the present analysis suggest the need for special treatment of transition strides between level walking and stair walking, rather than simply switching from one steady-state mode to the next. Importantly, our results show that the strategies adopted to facilitate these transitions should treat each of the four transition types as its own special case. For example, the stance phase ankle trajectories in three of the four transition types would be sufficiently approximated by the preceding mode, which also supports the finding that delaying the transition between walk and stair modes in a powered prosthesis largely does not affect the user^18^. However, this may not hold for stair ascent to level walking, where there is unique dorsiflexion seen in early stance. It has been suggested that appropriately-timed dorsiflexion of a device during stair ascent could help with vertical and forward propulsion of the leg as seen in able-bodied studies^36,39^.

The ankle joint in particular shows the most unique kinematics throughout these tasks, and given its function as the last link in the kinematic chain before contact with the environment, much of the challenge in commanding a prosthesis to safely emulate natural locomotor transitions lies here. In ankle angle trajectories, not only are there differences in the amplitude of local maxima and minima between SW, TR, and LW, but there are differences in the concavity of the trajectory. Still, while foot position is most directly altered by the ankle joint, the knee and hip cannot be discounted. One significant concern is how drastically an amputee’s hip trajectories will be affected by the weight and inertia of the prosthesis, particularly in the case of a transfemoral amputation. While one solution is to engineer the device to command excessive foot clearance, the inertial effects of such exaggerated motions could perturb balance. We contend that by aiming to emulate the unique transition kinematics of able-bodied persons, a more natural gait can be achieved.

### Gaussian process regression

Gaussian process regression models were trained across subjects for each joint to predict its angle using two categorical predictors (stride type, direction), and two continuous predictors (stair inclination angle, gait phase). RMSE results (Fig. 5) from leave-one-stride-out cross-validation indicated that average prediction error was between 4° and 8°. This demonstrates the usefulness of the GPR in that a single model can predict joint angles reasonably well when supplied with disparate data about the environment and ambulation context. As environment recognition continues to improve^20^, use of both categorical and continuous mechanical data in device control will become more ingrained in device control. The model was also not sensitive to the type of stride undertaken as we had hypothesized, with only one example of statistically significant ($p<0.05$) difference in error due to stride type. Surface fitting using GPR models (Fig. 4) demonstrated how a smooth surface can be used to represent continuous changes in terrain with relatively low errors in the predicted joint angles. This kind of model could be used to generate lookup tables for joint trajectories in controllers that account for the severity of terrain. The surfaces also provide visual insight into the complex effects of both terrain severity and stride type on joint trajectories. We hypothesized that the steady-state models would predict joint trajectories with lower error than the transition models because transitions pose fewer physical constraints to limb motion, and therefore allow for greater variability between subjects. Statistical analysis showed that there were only significant differences in error between tasks for the ankle joint during stair ascent.

## Conclusions

Our analyses show that among the four types of transitions between level walking and stair walking, there are numerous instances of joint trajectories not seen in either of the steady-state modes, and that these unique trajectories may occur at different parts of the gait cycle or in different joints depending on which transition type is being executed. This study encourages efforts to handle transition strides as more than a timed switching between steady-state modes of ambulation. We also report where each joint is significantly affected by differences in the inclination angle of the stairs. Finally, we show that Gaussian process regression models can be trained on all of these factors and across subjects to predict joint angles reasonably well, a possible way to generate reference trajectories for the control of powered assistive devices.

## Limitations

**Figure 6:**
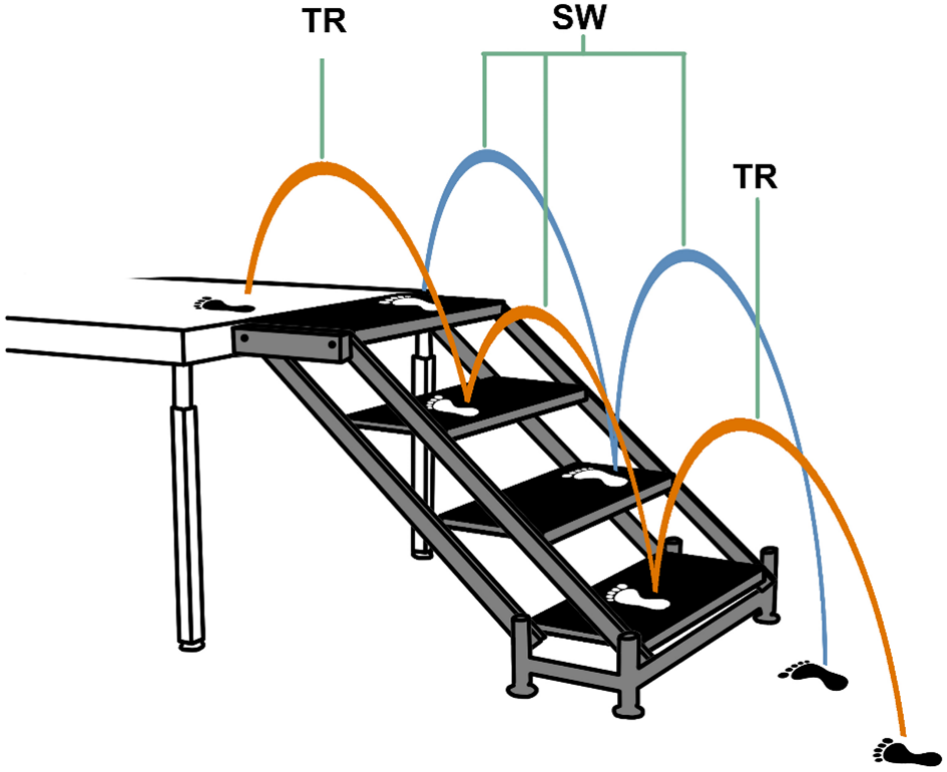
An adjustable staircase was used and strides were defined as shown. TR strides are those which only ascend or descend the height of a single step. Feet in this picture are oriented to depict stair ascent, but the TR and SW strides are defined the same way in either direction.

We were unable to control for step width/riser height due to the single adjustable staircase. Due to limited motion capture space, used a treadmill to gather steady-state level walking data, which has measurable effects on gait kinematics and kinetics^40–42^. Studies note, however, that the magnitudes of these differences are within the range of repeatability^41^ and that overall patterns are preserved between treadmill and overground walking^42^. Finally, the 4-step staircase meant that the we accepted all strides fully on the stairs as “steady-state” (Fig. 6). While it has been shown that this may not be enough steps to reach a steady-state velocity during stair descent^33^, our purely-kinematic analysis is not as concerned with velocity as a kinetic analysis, and velocity was expected to be variable due to inter-subject preferences. Future studies should investigate the development of steady-state walking mechanics over much larger staircases than the typical laboratory setups that include 3–6 stairs.

## Methods

### Experimental protocol

Ten able-bodied subjects (5 male, 5 female, 19–59 years, 53.7–87.0 kg) participated in the study after providing written informed consent. All methods in the study were approved by, and performed in accordance with, the relevant guidelines and regulations set forth by the UT Dallas Institutional Review Board. A custom set of 42 retroreflective markers was placed on anatomical landmarks of each subject’s lower body in order to obtain 3-dimensional kinematics using a ten-camera motion capture system at 100 Hz (Vicon Motion Systems, Oxford, UK) and Vicon’s Plug-in Gait pose estimation software. Subjects were then asked to approach and ascend, and approach and descend a four-step set of stairs at a comfortable pace, using a step-over-step strategy (i.e., without skipping stairs or stepping on any one stair with both feet^43^). Allowing approach to the steps at a comfortable pace rather than starting from rest at the first step was important for capturing the transitionary biomechanics we seek to investigate^44^. Subjects repeated ascending and descending tasks five times each with the stairs set at incline angles of 20°, 25°, 30°, and 35°, for a total of 20 ascents and 20 descents. The International Residential Code^9^ stipulates a maximum riser height of 7.75″ and minimum tread depth of 10″, corresponding to an incline angle of 37.8°, which is approximated by our steepest setting of 35°. Level walking data was collected from one minute of walking on a treadmill at 1 m/s once the steady pace had been achieved. The speed was fixed, as the data was recorded as part of a study where walking speed was controlled for across subjects^28^. While 1 m/s is relatively slow for the average adult walking speed^45^, this speeds is more inclusive of elderly and amputee populations, and helps reflect the slow-down observed in anticipation of encountering stairs^26^. A portion of this raw dataset is available at^28^.

### Data processing

Raw trajectories were low-pass filtered (4th order Butterworth, 6 Hz cutoff) and used as inputs to an inverse kinematic model to estimate ankle, knee, and hip joint kinematics in Nexus 2.8 (Vicon Motion Systems, Oxford, UK). A custom Matlab pipeline (Mathworks, Natick, MA, USA) was implemented to parse and time-normalize individual strides. Strides were classification into three types: level walking (LW), which came from treadmill walking; transitions (TR), which include the two strides that ascend/descend the height of a single step; and stair walking (SW), which was defined as the three strides that ascend/descend the height of two steps. It should be noted that this classification scheme counts the trailing leg transition stride as steady-state because it is based purely on the vertical displacement of the stride. Figure 6 depicts the staircase and which strides were considered TR and SW.

### Statistical analysis

Each subject’s data were vetted for outliers, and then averaged to get one subject-representative ankle, knee, and hip trajectory for each stride type. These subject-representative strides were then used as the inputs for statistical tests. For every inclination angle and in both directions, the TR trajectories were compared against the LW and SW trajectories using statistical parametric mapping (SPM). SPM was carried out using the *spm1d* (spm1d.org) package in Matlab. SPM allows the generalization of classical statistical tests to time series data, so that *regions* of significant difference in trajectories instead of singular points of interest can be investigated^46^. For transition analysis, paired t-tests across the ten subjects were conducted between LW and TR, and then between TR and SW, with $\alpha =0.95$ and a Bonferroni correction for multiple comparisons. Paired t-tests were chosen over ANOVA to investigate transition behavior because we are interested in the pairwise comparison of TR strides to LW and SS, but not the comparison of LW to SS, as these are different terrains entirely. Due to insufficient motion capture data, subjects 2, 6, 9, and 10 were omitted from the stair ascent to level walking transition.

For the effects of inclination angle, a 1-way ANOVA was performed across the four inclinations, and significant main effects ($p<0.05$) were reported.

### Regression models

For each joint, a Gaussian process regression (GPR) model was trained on strides from all subjects for each inclination angle and task, with the goal of predicting the average joint angles for a given ambulation context (gait phase, stair incline, stride type, and direction). This resulted in a total of 3 GPRs performed to model the ankle, knee, and hip, based on all subjects pooled together. A GPR is a nonparametric, kernel-based probabilistic model^32^ that was chosen here over traditional linear regression due to the complexity of the gait trajectories. The model ${\varvec{f}}({\varvec{x}})$ is a distribution over Gaussian functions with mean function ${\varvec{m}}({\varvec{x}})$ and covariance function ${\varvec{k}}({\varvec{x}},{\varvec{x}}\boldsymbol{^{\prime}})$, which chosen as the squared exponential function.

$${\varvec{f}}\left({\varvec{x}}\right) \sim {\varvec{G}}{\varvec{P}}\left({\varvec{m}}\left({\varvec{x}}\right),{\varvec{k}}\left({\varvec{x}},{\varvec{x}}\boldsymbol{^{\prime}}\right)\right)$$

$${\varvec{k}}\left({\varvec{x}},{\varvec{x}}\boldsymbol{^{\prime}}|{\varvec{\theta}}\right)={\sigma }_{f}^{2}\mathrm{exp}\left[-\frac{1}{2}\frac{{\left({\varvec{x}}-{\varvec{x}}\boldsymbol{^{\prime}}\right)}^{T}\left({\varvec{x}}-{\varvec{x}}\boldsymbol{^{\prime}}\right)}{{\sigma }_{l}^{2}}\right]$$

The mean function is the expected value of the posterior distribution of possible functions, and is used to make predictions. The covariance function is used to kernelize the prior functions, where ${\sigma }_{f}$ is the signal standard deviation, ${\sigma }_{l}$ is the characteristic length scale, and ${\varvec{\theta}}$ is a hyperparameter based on ${\sigma }_{f}$ and ${\sigma }_{l}$.

Leave-one-stride-out cross validation was used to obtain statistics on the root mean square error (RMSE) for each of the joint models during transitions and steady-state stair walking. A one-way ANOVA was performed to determine significantly ($p<0.05$) differing errors in both the ascending and descending tasks. When significance was encountered, pairwise comparisons were made using the Tukey–Kramer method.

## Data Availability

A portion of these raw data have been made available previously^28^. To access our dataset and mathematical models, we are happy to consider reasonably-made requests. Please contact the corresponding author, Ross M. Neuman, for such inquiries.

## References

[1] Nagata H (1991). Analysis of fatal falls on the same level or on stairs/steps. Saf. Sci..

[2] Friedland D, Brunton I, Potts J (2014). Falls and traumatic brain injury in adults under the age of sixty. J. Community Health.

[3] Abolhassani F, Moayyeri A, Naghavi M, Soltani A, Larijani B, Shalmani HT (2006). Incidence and characteristics of falls leading to hip fracture in Iranian population. Bone.

[4] Blazewick DH, Chounthirath T, Hodges NL, Collins CL, Smith GA (2018). Stair-related injuries treated in United States emergency departments. Am. J. Emerg. Med..

[5] Lythgo N, Begg R, Best R (2007). Stepping responses made by elderly and young female adults to approach and accommodate known surface height changes. Gait Posture.

[6] Lee HJ, Chou LS (2007). Balance control during stair negotiation in older adults. J. Biomech..

[7] Reid SM, Graham RB, Costigan PA (2010). Differentiation of young and older adult stair climbing gait using principal component analysis. Gait Posture.

[8] Chiu SL, Chang CC, Dennerlein JT, Xu X (2015). Age-related differences in inter-joint coordination during stair walking transitions. Gait Posture.

[9] “SECTION R311 MEANS OF EGRESS,” in *2021 International Residential Code*, Country Club Hills, IL: ICC, 2021, p. R311.7 Stairways.

[10] Jacobs JV (2016). A review of stairway falls and stair negotiation: Lessons learned and future needs to reduce injury. Gait Posture.

[11] Blanchet R, Edwards N (2018). A need to improve the assessment of environmental hazards for falls on stairs and in bathrooms: Results of a scoping review. BMC Geriatr..

[12] Templer, J., Mullet, G. M., Archea, J. & Margulis, S. T. An analysis of the behavior of stair users, 1–74 (1978) [Online]. https://nvlpubs.nist.gov/nistpubs/Legacy/IR/nbsir78-1554.pdf. Accessed 16 Nov 2020.

[13] Culver S, Bartlett H, Shultz A, Goldfarb M (2018). A stair ascent and descent controller for a powered ankle prosthesis. IEEE Trans. Neural Syst. Rehabil. Eng..

[14] O. A. Kannape & H. M. Herr. Volitional control of ankle plantar flexion in a powered transtibial prosthesis during stair-ambulation. in *2014 36th Annu. Int. Conf. IEEE Eng. Med. Biol. Soc. EMBC 2014*, 1662–1665 (2014). 10.1109/EMBC.2014.6943925. 10.1109/EMBC.2014.6943925 25570293

[15] Nakamura BH, Hahn ME (2017). Myoelectric activation pattern changes in the involved limb of individuals with transtibial amputation during locomotor state transitions. Arch. Phys. Med. Rehabil..

[16] Au S, Berniker M, Herr H (2008). Powered ankle-foot prosthesis to assist level-ground and stair-descent gaits. Neural Netw..

[17] Rabe KG, Fey NP (2022). Evaluating electromyography and sonomyography sensor fusion to estimate lower-limb kinematics using gaussian process regression. Front. Robot. AI.

[18] Simon AM (2017). Delaying ambulation mode transition decisions improves accuracy of a flexible control system for powered knee-ankle prosthesis. IEEE Trans. Neural Syst. Rehabil. Eng..

[19] B. Laschowski, W. McNally, A. Wong, & J. McPhee. Computer vision and deep learning for environment-adaptive control of robotic lower-limb exoskeletons. in *Annu. Int. Conf. IEEE Eng. Med. Biol. Soc. IEEE Eng. Med. Biol. Soc. Annu. Int. Conf.*, Vol. 2021, 4631–4635 (2021). 10.1109/EMBC46164.2021.9630064. 10.1109/EMBC46164.2021.9630064 34892246

[20] Laschowski B, McNally W, Wong A, McPhee J (2022). Environment classification for robotic leg prostheses and exoskeletons using deep convolutional neural networks. Front. Neurorobot..

[21] D. H. Gates, J. Lelas, U. Della Croce, H. Herr, & P. Bonato. Characterization of ankle function during stair ambulation. in *Annu. Int. Conf. IEEE Eng. Med. Biol.—Proc.*, vol. 26 VI, 4248–4251 (2004). 10.1109/iembs.2004.1404184. 10.1109/IEMBS.2004.1404184 17271242

[22] Cheng S, Bolivar-Nieto E, Welker CG, Gregg RD (2022). Modeling the transitional kinematics between variable-incline walking and stair climbing. IEEE Trans. Med. Robot. Bionics..

[23] Mcfadyen BJ, Winter D (1988). An integrated biomechanical analysis of normal stair ascent and descent. J. Biomech..

[24] Livingston LA (1991). Stairclimbing kinematics on stairs of differing dimensions. Arch. Phys. Med. Rehabil..

[25] Riener R, Rabuffetti M, Frigo C (2002). Stair ascent and descent at different inclinations. Gait Posture.

[26] Peng J, Fey NP, Kuiken TA, Hargrove LJ (2016). Anticipatory kinematics and muscle activity preceding transitions from level-ground walking to stair ascent and descent. J. Biomech..

[27] Sheehan RC, Gottschall JS (2011). Stair walking transitions are an anticipation of the next stride. J. Electromyogr. Kinesiol..

[28] Reznick E, Embry KR, Neuman R, Bolívar-Nieto E, Fey NP, Gregg RD (2021). Lower-limb kinematics and kinetics during continuously varying human locomotion. Sci. Data.

[29] Yun Y, Kim HC, Shin SY, Lee J, Deshpande AD, Kim C (2014). Statistical method for prediction of gait kinematics with Gaussian process regression. J. Biomech..

[30] Hong J, Chun C, Kim SJ, Park FC (2019). Gaussian process trajectory learning and synthesis of individualized gait motions. IEEE Trans. Neural Syst. Rehabil. Eng..

[31] J. Zhang, H. An, Y. Huang, Q. Wei, & H. Ma. Flat-upstairs gait switching of lower limb prosthesis via Gaussian process and improved Kalman filter. in *2021 6th IEEE Int. Conf. Adv. Robot. Mechatronics, ICARM 2021*, 241–244 (2021). 10.1109/ICARM52023.2021.9536178.

[32] Rasmussen CE, Williams CKI (2006). Gaussian Processes for Machine Learning.

[33] Cluff T, Robertson DGE (2011). Kinetic analysis of stair descent: Part 1. Forwards step-over-step descent. Gait Posture.

[34] Costigan PA, Deluzio KJ, Wyss UP (2002). Knee and hip kinetics during normal stair climbing. Gait Posture.

[35] Lin YC, Fok LA, Schache AG, Pandy MG (2015). Muscle coordination of support, progression and balance during stair ambulation. J. Biomech..

[36] Harper NG, Wilken JM, Neptune RR (2018). Muscle function and coordination of stair ascent. J. Biomech. Eng..

[37] Prentice SD, Hasler EN, Groves JJ, Frank JS (2004). Locomotor adaptations for changes in the slope of the walking surface. Gait Posture.

[38] McFadyen BJ, Carnahan H (1997). Anticipatory locomotor adjustments for accommodating versus avoiding level changes in humans. Exp. Brain Res..

[39] Harper NG, Wilken JM, Neptune RR (2018). Muscle function and coordination of amputee stair ascent. J. Biomech. Eng..

[40] Chang MD, Shaikh S, Chau T (2009). Effect of treadmill walking on the stride interval dynamics of human gait. Gait Posture.

[41] Riley PO, Paolini G, Della U, Paylo KW, Kerrigan DC (2007). A kinematic and kinetic comparison of overground and treadmill walking in healthy subjects. Gait Posture.

[42] S. J. Lee & J. Hidler. Biomechanics of overground vs. treadmill walking in healthy individuals. **104**, 747–755 (2007). 10.1152/japplphysiol.01380.2006. 10.1152/japplphysiol.01380.2006 18048582

[43] Reid SM, Lynn SK, Musselman RP, Costigan PA (2007). Knee biomechanics of alternate stair ambulation patterns. Med. Sci. Sport. Exerc..

[44] Vallabhajosula S, Yentes JM, Momcilovic M, Blanke DJ, Stergiou N (2012). Do lower-extremity joint dynamics change when stair negotiation is initiated with a self-selected comfortable gait speed?. Gait Posture.

[45] Charalambous CP (1989). Repeatability of kinematic, kinetic, and electromyographic data in normal adult gait. Class. Pap. Orthop..

[46] Pataky TC, Vanrenterghem J, Robinson MA (2015). Zero- vs. one-dimensional, parametric vs. non-parametric, and confidence interval vs. hypothesis testing procedures in one-dimensional biomechanical trajectory analysis. J. Biomech..

